# News and Notes

**Published:** 1901-02

**Authors:** 


### *NEWS AND NOTES.*

DR. E. H. RICHARDSON has been elected a member of the Atlanta Board of Health.

THE death is reported of Dr. J. N. Huffaker of Plainville, Ga., following an operation for tumor.

AN exchange says: "Corning's cord cocainization has come to the front." We were under the impression that it was always behind.

THE inventor of the laryngoscope, says the *Medical Times*, is a Spaniard of Madrid named Manuel Garcia. He is now ninety-five years old.

AT a recent meeting of the Atlanta Board of Health, Dr. W. C. Jarnigan was re-elected President. Dr. E. H. Richardson succeeds Dr. C. F. Benson as Secretary.

"DR." C. R. KING of the New Medicine Co. of Atlanta has been fined $100 by Judge Newman of the federal court for sending obscene matter through the mails.

DR. W. C. FISHER has been elected to the office of county physician for Fulton county vice Dr. B. W. Bezzell, resigned. The latter retains his position on the Board of Health.

COUNCILMAN DR. T. D. LONGINO proposes a substitute for the present system of seven ward physicians, at a salary of $50 a month each, a health officer with the requisite number of assistants.

WE regret to announce the death of Dr. T. B. Wheeler of Montreal, Canada, the originator of the well-known Wheeler's

Tissue Phosphates. THE JOURNAL-RECORD extends its condolences.

---

THE reports of an outbreak of the plague at Vladivostock in Russia are confirmed. There have been 19 cases, of which 15 were fatal. Four plague patients are still in the hospital and numbers are isolated.

---

SOME time ago an institution was opened in Belgium for the purpose of carrying out a cure for tuberculosis by an exclusive raw meat diet. After an existence of not quite three months the experiment has been abandoned.

---

*New York State Journal of Medicine* is the title of a new monthly periodical to be devoted to the interests of the New York State Medical Association. The chairman of its editorial committee is Dr. James Hawley Burtenshaw.

---

THE heirs and legatees of Dr. T. W. Evans have reached an agreement to have the estate settled in Philadelphia. About $3,000,000 will become at once available for the construction and foundation of the dental institute in that city.

---

A CONSOLIDATION of two important medical colleges in St. Louis under the name of the Marion Sims-Beaumont College of Medicine, which was effected November 22, is said to be preliminary to affiliation with the St. Louis University as its medical department.

---

THE Johns Hopkins Hospital will be defendant in a suit of damages for $5,000 which has been brought against that institution by two individuals who claim to have received personal injuries through a live electric wire while walking along the street in front of the hospital.

THE following gem is taken from the *Cleveland Medical Gazette:*

"Mamma, what's twins?" asked the smallest child.

"I know," replied the older one, before the mother could answer. "Twins is two babies just the same age. Three babies are triplets, four are quadrupeds, and five are centipedes."

---

DIAGNOSED.—Della—Orlando Smiggs was out to see me last night, and he was, oh, so nervous, and showed so plainly that he had something on his mind that I was sure he was going to propose, but he couldn't muster up the courage.

Ophelia—Yes, Orlando is subject to those sudden attacks of cholera morbus.

---

DR. RICHARD BEVERLY COLE died at San Francisco, aged 72 years. He will be pleasantly remembered by the local profession as the President of the American Medical Association during its meeting at Atlanta in 1896. Dr. Cole was a native of Virginia, and had long been one of the notable figures in the medical profession of the western section of the country.

---

M. VAILLANT'S resolution has passed the Chamber of Deputies, calling on the French Government to prohibit the manufacture and sale of all alcoholic liquors which shall be pronounced dangerous by the Academy of Medicine. The consumption of absinthe has reached 10,000,000 liters annually, and it is against this destructive intoxicant that the resolution is directed.

---

E. H. PLUMACHER, United States Consul at Maracaibo, has sent the following report to the State Department: "A simple remedy against mosquitoes has been employed in several places in South America and is equally well adapted to the temperate zone. It consists in planting the castor-oil plant around the house and premises." This is an old and exploded idea.

ACCORDING to the report of the Marine Hospital Service there are a large number of cases of enteric fever prevalent in Galveston, Texas. One can only estimate them, for there is no ordinance that requires them to be reported to the Board of Health. Dr. William Fischer, former health officer, estimated the number about 450. There are six cases of smallpox and a considerable number of diphtheria and scarlatina.

---

A BILL for the protection of physicians and surgeons from blackmail and unjust malpractice suits is also to be introduced this session. Its most important provision will be the requirement of a bond from any one bringing a suit against a physician for alleged malpractice. This bond is to be forfeited in case the judgment goes against him. This idea is based on a somewhat similar provision in English law.—*Medical News*.

---

AT a recent meeting of the Chicago Medical Society a resolution was offered and adopted that pathological specimens, which are now taxed at 20 per cent. on the cost of their production, should be admitted duty free, as they are used exclusively for scientific purposes and are of no commercial value. A copy of the resolution was ordered to be transmitted to each Senator from Illinois, and each member of Congress from this city.

---

DR. DE MOURA of Brazil, recently contributed a paper to the Berlin Medical Society on the result of his researches and observations on the cure of leprosy by the inoculation of rattlesnake poison. He claims that if the patient be suffering from uncomplicated leprosy the treatment is followed by excellent results; and Professor Lewin, while he does not admit that the venom is of the nature of an antidote, acknowledges that it produced at any rate a temporary change for the better, and is, therefore, worthy of further investigation.

A DOCTOR once presented himself at the Golden Gates for admission, and after passing a fair examination as to his conduct, Saint Peter agreed to permanently admit him if he could pick out Adam and Eve from the assembled angels. The doctor looked around and soon found his progenitors. But Peter was puzzled, and asked the doctor how in the name of the golden harps had he managed to recognize the first couple. "Oh!" said the doctor, "that is quite easy; they are the only ones without an umbilicus." —*Indian Lancet.*

---

A RECENT newspaper account of an operation performed under the anesthetic influence of hypnotism relates that the "patient was an interested spectator," and watched the "warm blood spurt under the surgeon's knife!" As the operation in question was a curettement of the uterus it would be exceedingly interesting to know just how the lady managed to see all this. Even the proverbial curiosity of the sex would find it difficult, we should think, to overcome anatomical difficulties in the way of observation, which might appall even a "rubber woman."—*Medical Standard.*

---

THE following is an exact copy of an epitaph on a tombstone in a New Hampshire cemetery. It explains itself:

"Ruth Sprague, dau. of Gibson and Elizabeth Sprague. Died Jan. 11, 1816; aged 9 years, 1 mos. and 3 days.

"She was stolen from the grave by Roderick R. Clow and dissected at Dr. P. M. Armstrong's office, in Hoosick Falls, from which place her mutilated remains were obtained and deposited here.

"Her body dissected by fiendish men,
Her bones anatomized,
Her soul we trust has risen to God,
A place where few physicians rise."

PROFESSOR CELLI, who is a member of the Italian Parliament, will, it is said, present to the Chamber a drastic proposal for the suppression and prevention of malaria in Italy. He proposes to make punishable by law the neglect of landowners and all employers of labor to provide in malarial districts every means of fighting the fever. Signori Boselli and Sonnino have submitted to the President of the Chamber a proposal that the Minister of Finance be empowered to purchase a supply of quinine of the purest quality, which is to be sold to the public at a trifle over cost price, the margin being intended to protect the government against loss from fluctuations in the price of the raw material. It is calculated that the very slight profit on the sale would average $200,000 yearly, which would be devoted to the extermination of malaria. This proposal, it is said, is likely to meet with formidable opposition.

---

ACTING on the recommendations of the Board of Officers Governor-General Wood has issued an order in Havana which declares that the chief surgeon of the Department of Cuba has reported that it is now believed to be well established that malaria, yellow fever and filarial infection are transmitted by the bites of mosquitoes. The troops are enjoined to observe carefully two precautions: First, they are to use mosquito bars in all barracks, hospitals and on field service whenever practicable; second, they are to destroy the "wigglers" or young mosquitoes by the use of petroleum on the waters where they breed. Permanent pools or puddles are to be filled up. To the others are to be applied one ounce of kerosene to each fifteen square feet of water, twice a month, which will destroy not only the young, but the old mosquitoes. This does not injure drinking-water if drawn from below and not dipped out. Protection is thus secured, according to the order, because the mosquito does not fly far, seeks shelter when the wind blows and that each community breeds its own mosquitoes.—*Medical News.*

